# Supplementary material for: Targeting the Exon2 splice cis-element in PD-1 and its effects on lymphocyte function
Source: PLoS One. 2025 Sep 8;20(9):e0331468. doi: 10.1371/journal.pone.0331468 (PMC12416725; doi:10.1371/journal.pone.0331468)
Supplement: S1 Table — (PDF) [file pone.0331468.s001.pdf]

Supplementary Information-1

Guide name	Sequence (5’-3’)
guide1	ctgtctggggagtctgagagatggagag
guide2	tctttgatctgcgccttgggggccaggg
guide3	ggacagagccctggactggagctggggg
guide4	ggggtgcttccagagctagaggacagag
guide5	tctgcactgctctggcacaggggagtgt
guide6	ccacgagcagggctggggagaaggtggg
guide7	agaggtgaggaaggggctgggtggcccc
guide8	ggagaagctgcaggtgaaggtg
guide9	ggcctccgaggccgcacctgtcaccctg
guide10	ccactgcagagcctcttccttctacgtg
guide11	tccttctacgtgaggctgcagcttctgc
guide12	cccggccacctgctcacatccctcgggc
guide13	tgggtggccccacaaagcctccccggcc
guide14	gagtctgagagatggagagaggtgagga
guide15	cgcacctgtcaccctgagctctgcccgc
guide16	tcagctcacccctgccccggggcctccg
guide17	gaggggacacccaccccaggaccggctc
guide18	gagctcctgatcctgtgcaggaggggac
