# Supplementary material for: Targeting the Exon2 splice cis-element in PD-1 and its effects on lymphocyte function
Source: PLoS One. 2025 Sep 8;20(9):e0331468. doi: 10.1371/journal.pone.0331468 (PMC12416725; doi:10.1371/journal.pone.0331468)
Supplement: S2 Table — (PDF) [file pone.0331468.s002.pdf]

G-CSF		GM-CSF		Frakutalkin		IFN-g		IL-10		IL-13	
Non-targeting	Targeting	Non-targeting	Targeting	Non-targeting	Targeting	Non-targeting	Targeting	Non-targeting	Targeting	Non-targeting	Targeting
66.1	15.5	7468.9	4241.9	7.2	34.7	6984.4	1317.3	154.8	149.7	10514.3	10552.4
75.8	18.9	6900.3	4080.7	44.3	38.2	6215.2	1348.0	134.9	148.6	10555.5	10843.5
66.1	22.2	7057.0	4139.3	30.7	34.7	6798.1	1514.0	169.0	152.1	10704.3	10580.2
58.6	28.5	6899.1	4789.5	52.1	20.0	6722.1	1900.2	145.1	192.8	10581.1	10459.0
58.6	18.9	6817.1	4077.4	44.3	49.6	6035.2	1161.7	132.2	153.0	10489.2	10629.9
56.1	18.9	6047.5	4144.0	41.3	56.7	5276.2	1262.3	132.5	169.0	10709.6	10604.6
56.1	17.2	6524.8	3743.7	20.0	7.2	5875.3	1188.5	136.1	150.9	10426.2	10584.6
48.2	15.5	6377.4	3876.1	30.7	34.7	5768.6	1110.1	142.1	163.0	10655.7	10499.6
45.5	22.2	6257.9	3980.7	44.3	0.0	5149.7	1256.7	125.2	156.6	10418.5	10571.6
53.5	17.2	6114.8	3306.8	47.0	0.0	5825.3	1006.2	133.1	131.3	10568.1	10596.3
53.5	18.9	5778.3	3682.7	49.6	44.3	5063.2	1081.3	166.1	149.4	10636.0	10588.5
50.9	15.5	6033.7	3559.4	0.0	34.7	5289.2	987.4	123.2	125.6	10343.4	10575.9
50.9	18.9	7243.8	3509.9	20.0	44.3	5563.5	1035.5	132.5	141.8	10576.3	10518.6
56.1	18.9	5717.7	3645.1	34.7	30.7	5379.3	1069.0	143.6	123.5	10459.4	10546.4
68.6	15.5	6913.8	4076.1	30.7	20.0	6844.3	1284.5	128.3	153.0	10514.3	10491.8

IL-4		IL-5		IL-6		IL-8		CXCL10		TNF-alpha	
Non-targeting	Targeting	Non-targeting	Targeting	Non-targeting	Targeting	Non-targeting	Targeting	Non-targeting	Targeting	Non-targeting	Targeting
2533.8	1143.6	2701.8	2816.8	61.0	15.6	3766.9	3283.6	13073.3	12929.2	2349.9	624.5
1874.5	1229.3	2835.6	2334.2	53.0	15.8	3662.2	3341.7	12971.2	12890.1	2055.0	587.0
2305.3	1212.4	2793.2	2454.5	65.0	22.8	3653.7	3347.7	12810.1	12972.6	2221.6	629.8
2514.2	1482.3	2599.0	3305.7	66.8	23.7	3643.0	4164.5	12988.8	13269.9	2183.4	784.8
2359.7	1058.5	2649.9	2410.6	58.0	14.7	2593.0	2479.5	12709.3	13110.2	2008.7	572.9
2178.3	1052.6	2951.3	2739.0	56.1	19.1	2912.9	3015.4	12611.2	12956.3	1934.5	580.8
2079.9	995.0	2332.5	2417.1	59.1	14.0	2804.9	2691.1	12867.9	13080.1	1874.0	578.6
2072.2	1197.8	2657.6	2487.5	56.9	15.6	2843.9	2393.7	12682.7	12880.7	1850.1	559.5
2091.6	1168.1	2215.8	2388.2	57.1	14.2	2661.7	2801.5	12653.6	13035.1	1903.5	550.7
2132.3	929.1	2569.1	2260.6	48.9	15.7	2737.7	2157.2	12518.4	12788.7	1939.5	420.2
1814.0	1072.5	2563.2	2227.9	58.4	16.6	2534.9	2407.3	12504.6	12576.9	1873.4	541.2
1964.5	1158.7	2645.8	2135.8	47.1	16.9	2577.3	2282.0	12693.4	12828.2	1908.9	462.5
1945.6	1010.7	2810.1	2610.8	49.4	11.3	2627.0	2203.3	12898.9	13020.1	2029.0	531.2
1772.9	1130.2	2362.0	2509.0	52.7	14.5	1998.9	2196.5	12684.1	12746.6	1759.9	540.2
2026.8	1287.7	2588.4	2181.7	44.8	13.1	2424.1	2464.4	12910.3	12707.3	1983.3	591.2
